# The earliest cotton fibers and Pan-regional contacts in the Near East

**DOI:** 10.3389/fpls.2022.1045554

**Published:** 2022-12-08

**Authors:** Li Liu, Maureece J. Levin, Florian Klimscha, Danny Rosenberg

**Affiliations:** ^1^ Department of East Asian Languages and Cultures, Stanford University, Stanford, CA, United States; ^2^ Stanford Archaeology Center, Stanford University, Stanford, CA, United States; ^3^ School of Human Inquiry, University of Arkansas at Little Rock, Little Rock, AR, United States; ^4^ Archaeology Division, Department of Research/Collection, Lower Saxony State Museum, Willy-Brandt-Allee 5, Hannover, Germany; ^5^ Laboratory for Ancient Food Processing Technologies (LAFPT), Zinman Institute of Archaeology, University of Haifa, Haifa, Israel

**Keywords:** microbotanical analysis, cotton, bast fibers, dyed fibers, chalcolithic, tel tsaf, jordan valley

## Abstract

Fiber technology (cordage and textile) has played a central role in all human societies for thousands of years, and its production, application and exchange have deep roots in prehistory. However, fiber remains have only rarely been observed in prehistoric sites because they tend to decay quickly in normal environmental conditions. To overcome preservation problems of macroscopic remains, we employed microbotanical analysis on soils from anthropogenic sediments in activity areas at Tel Tsaf in the Jordan Valley, Israel (ca. 5,200–4,700 cal BC), and recovered fiber microremains. This includes at least two types of bast fibers and the earliest evidence of cotton in the Near East, some of which were dyed in various colors. Some of these fibers likely represent the remnants of ancient clothing, fabric containers, cordage, or other belongings. The cotton remains, probably derived from wild species originating in South Asia, predate the oldest known cotton domestication in the Indus Valley by about two millennia. Tel Tsaf played a pivotal role in trans-regional trade and exchange networks in the southern Levant, and the presence of cotton at the site points to possible connections with the Indus Valley as early as 7,200 years ago.

## 1 Introduction

Fiber technology (including cordage and textiles) has played a central role in human societies for tens of thousands of years, and its production, application, trade, and exchange have deep roots in prehistory (Barber, 1991; Gilligan, 2010; Sabatini and Bergerbrant, 2020; Schier and Pollock, 2020). This was recently exemplified by the rare find of a 46,000-y-old cord fragment made from inner bark fibers at the Neanderthal site of Abri du Maras in France (Hardy et al., 2020). However, cordage, and particularly textiles, have often been described as an invisible technology or “the missing majority” (Hurcombe, 2014) in archaeology because they tend to decay quickly in normal environmental conditions. Since the remains of actual prehistoric textiles are rarely found, it is often through a synthesis of available secondary evidence (e.g., texts, tools, and agrarian and pastoral practices) that textile production and exchange are reconstructed (McCorriston, 1997; Nosch et al., 2013). This preservation problem severely impacts our knowledge about the history of textiles in terms of their origins, materials, production, exchange, and usage.

In the ancient Near East, the earliest cord fragments (23,000 cal BP), made from a monocotyledonous plant, were recovered from the Upper Paleolithic site of Ohalo II in Israel (Nadel et al., 1994), but textile remains appeared in the archaeological record much later. The earliest and most used indigenous materials for producing textiles were local bast fibers, from plants such as flax, (Zohary et al., 2012; Bar-Yosef, 2020), and the bark of oak trees, which has been identified at Çatalhöyük, dating to ca. 8,500 years ago (Rast-Eicher et al., 2021). Cotton as a non-indigenous fiber was introduced to the Near East, and the earliest known cotton remains were reported from Dhuweila in Badia of eastern Jordan, dating to the Late Chalcolithic or Early Bronze Age (4,450-3,000 BC) (Betts et al., 1994). Production of wool from domesticated sheep was part of the Secondary Products Revolution (Sherratt, 1983). Some scholars believe that this technology may have been developed around the 4^th^ millennium BC during the Late Chalcolithic period (Sherratt, 1983; Becker et al., 2016), while others have argued that wool processing developed as early as 7,000 BC in Mesopotamia as the first fiber of prestige before becoming more widely available (Breniquet, 2020).

Given that findings of macroscopic textiles are extremely scarce in prehistoric sites, the current archaeological record is insufficient for understanding textile production and use. This paucity is mainly a methodological problem, and more techniques are needed to help identify textiles and fibers from diverse archaeological contexts. Several recent studies based on micro-remain analysis have proven effective in recovering some “invisible” fibers and textiles in various archaeological contexts. The earliest plant microfibers on stone tools, although not necessarily related to tool function, have been recovered at the Paleolithic site of Schöningen in Germany, dating to ca. 300,000 BP (Rots et al., 2015). Bast microfibers, perhaps for making cords, waving baskets, or sewing garments, have been found in 30,000-year old Upper Paleolithic deposits at Dzudzuana Cave, Georgia (Kvavadze et al., 2009; Kvavadze et al., 2010a). Abundant microfibers, predominantly bast but also wool, have been recovered in soil samples throughout the entire deposits of eight stratigraphic layers, measured 15 m in depth, at Locality 29 in the Shizitan site cluster, north China, dating to 28,000-13,500 cal. BP (Song et al., 2017). Residue analysis of boulder mortars from a Natufian site at Raqefet Cave, Israel, has also revealed bast microfibers, which were probably used to make bags or baskets around 13,000 years ago (Liu et al., 2018). A systematic analysis of microfossil remains on human calculus and burial site sediment samples from Luistari cemetery in Finland (ca. 600-1200 cal. AD) has recovered phytoliths, parasite eggs, bast and animal fibers, and feathers, when few other grave goods are preserved (Juhola et al., 2019). These findings demonstrate that microfibers can survive in anthropogenic sediments for a very long time and can be examined under light microscopes. The studies of such micro-particle remains, often described as a type of Non-Pollen Palynomorph (NPP), have demonstrated a great potential for the reconstruction of human activities (Henry, 2020; Shumilovskikh and Geel, 2020; O’Keefe et al., 2021). Following this line of investigation, we employed microbotanical analysis on sediment samples collected from the Middle Chalcolithic site at Tel Tsaf in Israel (ca. 5,200–4,700 cal. BC) to test for the presence of fibers.

## 2 Materials and methods

### 2.1 Archaeological background

**Figure 1:**
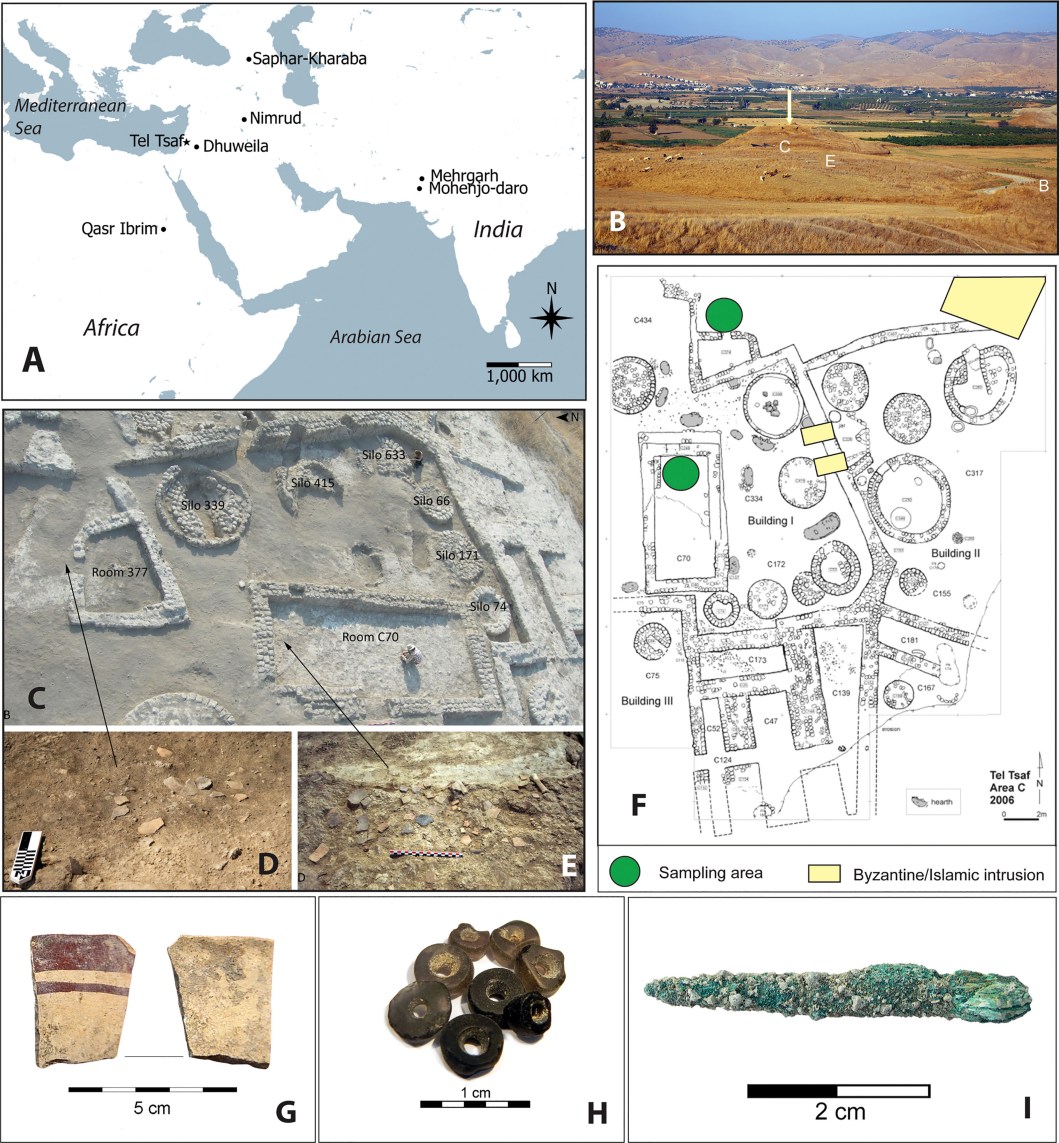
Site locations and major remains in Area C at Tel Tsaf. **(A)** Location of Tel Tsaf and sites associated with early cotton discussed in this study; **(B)** locations of Areas C, E, B at Tel Tsaf, viewed from the west (arrow pointing to the central high mound of Area C); **(C)** Room C70 and associated structures in Building CI (Courtesy of Y. Garfinkel); **(D, E)** sampling areas, showing pottery concentrations; arrows mark their approximate locations; **(F)** plant view of Area C, showing Middle Chalcolithic architecture, the areas where soil samples were taken for this study, and locations of late intrusions; **(G)** a Ubaid sherd found near Room C70; **(H)** obsidian beads found together in Room C70; **(I)** a copper awl found with Burial C555 near Room C70. **(F, I)** original image courtesy of Y. Garfinkel.

Tel Tsaf is located in the Middle Jordan Valley, Israel (**Figure 1A**). The site covers ca. five hectares and consists of three low hills that were formed by laminated layers of sediment deposited by the Pleistocene Lake Lisan (Garfinkel et al., 2020) (**Figure 1B**). The excavations in Area C, where the current samples were taken from, revealed the following stratigraphic sequence: (I) Meagre and scattered remains belonging to Late Byzantine to Early Islamic activities immediately below the site topsoil; (II) A sterile layer of fine yellowish sediment distributed over part of the site, probably deposited by wind, reaching over 1 m in thickness at the highest point of the tel; (III-IV) Middle Chalcolithic remains, ca. 2 m in thickness, characterized by massive, well-built, mudbrick rectilinear elements of buildings (Garfinkel et al., 2007; Garfinkel et al., 2020) (**Figures 1C-F**). The exceptional organic preservation at the site and its chronological location made this site a perfect candidate to study subsistence strategies during the Neolithic-Chalcolithic transition in the Near East (Rosenberg and Klimscha, 2021).

The site includes many types of objects indicative of long-distance contacts. This includes the earliest evidence for metallurgy in the southern Levant: a small copper awl found in the silo grave of a woman, its copper shown to be of non-local origin (Garfinkel et al., 2014). Other artifacts of distant origins include north Levantine Ubaid pottery and a clay stamped sealing; non-local figurines and beads, including Anatolian obsidian beads and other artifacts; and shells from the Nile River and Mediterranean Sea (Garfınkel et al., 2009; Freikman et al., 2021; Rosenberg and Klimscha, 2021) (**Figures 1G-I**). These non-local artifacts suggest that people at Tel Tsaf had connections with remote social groups, or at least engaged in trade with surrounding regions situated within long-distance networks. In addition, no artifactual evidence indicates that the site was associated with fabric production. Given its special nature, Tel Tsaf is an ideal locality to explore the possible early use and spread of various textiles as a new dimension in the development of long-distance trade in the Near East.

### 2.2 Methods

In the current study at Tel Tsaf, we collected 16 *in situ* soil samples from floors and under floors in Room C70 in Building CI as well as from surfaces north of Room C377, located just to north of and under the courtyard of Building CI, all from Area C. The sampling spots were about 1 m below the site’s surface. Although some scattered Byzantine/Islamic remains were found at site, they did not intrude on the sampling areas and no significant presence of later artifacts was found on top of the sampling areas (**Figure 1F**). It is also unlikely that groundwater activities could have caused significant downward movements of microfossil remains in this dryland environment with annual rainfall of ca. 270 mm (Israel Meteorological Service). Given these conditions, it is unlikely that microbotanical remains in these samples are primarily later intrusions, especially if they occur in high numbers and are large in size. To test for possible contamination, we collected four control samples from the topsoil at and around the site (**Table 1**).

**Table 1:** Tel Tsaf sample and fiber record.

Lab sample#	Sampling location	Bast	Cotton	Wool	UNID	Total
S1-1	L. 926, surface in Room C70, Bl. CI	1				1
S1-2		1	3		1	5
S2-1	L. 945, Room C70-under surface, Bl. CI	4		1	3	8
S2-2		6	3		6	15
S3-1	L. 930, surface 910, north of Room C377, located just to north of the courtyard of Building CI	4	6		4	14
S3-2		1			2	3
S 7-1	L. 965, surface in Room C70, Bl. CI	4	2		7	13
S7-2		1			2	3
S8-1	L. 965, surface in Room C70, Bl. CI	2	1		2	5
S8-2		18			4	22
S9-1	L. 965, surface in Room C70, Bl. CI	4			2	6
S9-2			2			2
S10-1	L. 930, surface 910, north of Room C377, located just north of the courtyard of Building CI			1	3	4
S10-2		7			1	8
S11-1	L. 930, surface 910, north of Room C377, located just to north of the courtyard of Building CI	5				5
S11-2		4			5	9
Total n		62	17	2	42	123
Total %		50.4	13.8	1.6	34.1	100.0
Ubiquity n		14	6	2	13	16
Ubiquity %		87.5	37.5	12.5	81.3	100.0
Control-1	The field at the foot of the Tel	1				1
Control-2	The Tel surface near southwest of Area C	2			1	3
Control-3	The Tel surface near west of Area C.	1			1	2
Control-4	About 1 km north of the site, near the road leading to the site	3				3
Control Total n		7			2	9

The soil samples from Building CI are regarded as remains of the living environment during the site occupation period. The soil samples are derived from two depositional contexts. One group are sediments from the interior surfaces of eight unwashed pottery sherds, including bowls, jars, and unidentified body fragments found *in situ*. Since the sherds may have been discarded after the pots were broken and remained in their depositional contexts for a long period of time, these sediment samples are likely to contain a mixture of residues derived from vessel use, local anthropogenic activities, and broader environmental vegetation in antiquity. The other group are eight soil samples near these sherds, which likely contained micro-remains from both anthropogenic contexts and the broader environment. They were labelled as -1 (on the sherd) and -2 (in the soil near the sherd) after the sample number (**Table 1**).

Pottery sherds were collected at the site and immediately wrapped in tin foil that was placed in a sealed plastic bag, and then the sediment on the interior surface of each sherd was scraped off with a clean metal blade and placed in tin foil in the Laboratory for Ancient Food Processing Technologies at the Zinman Institute of Archaeology, University of Haifa. The additional soil samples near the sherds were taken in the field and directly put in tin foil and sealed plastic bags. The sample collection processes were carried out with great care to prevent contamination in the field and the lab.

All the samples (each about 100 mg, and totaling about 1.6 g) were processed for microbotanical analysis in the Stanford Archaeology Center, Stanford University, with standard protocols. This involved two procedures: (1) EDTA (ethylenediaminetetraacetic acid; 0.1%) dispersion to release microparticles, such as fibers, from small soil microaggregates; and (2) SPT heavy liquid (sodium polytungstate, density 2.35) separation to extract microbotanical remains (Liu et al., 2018). Extracts were mounted in a 1:1 (vol:vol) solution of glycerol and distilled water on glass slides, covered with coverslips, and sealed with nail polish.

Previous studies have often employed SEM imaging for fiber identification, and the cross-section of fibers has been used as an essential variable for separating different fibers (Betts et al., 1994; Suomela et al., 2017; Lukesova and Holst, 2020). This method is highly effective if the observed objects are actual fibers or textiles. In our case, microfibers are found on sealed slides, thus it is not possible to use SEM method for observation. Nevertheless, researchers have demonstrated the high efficacy of polarized light microscopy for examining morphological features on plants used in textiles, achieving taxonomical identification (Paterson et al., 2017). A recent development in accurate identification of fibers is the modified Herzog test) also known as the red plate test). It helps determine different bast fibers by analyzing fibrillar orientation (either S-twist or Z-twist). Fibers oriented in angles at 0° and 90° were recorded for identification; the colors are expected to shift from blue at 0° to red or yellow at 90° for Z-twist fibers (in hemp and jute), and the expected color change is just opposite for S-twist fibers (in flax and nettle).This method also helps separate bast fibers from cotton, which does not have a well-defined fibrillar angle, thus will show rapid color change along the fiber (Bergfjord and Holst, 2010; Haugan and Holst, 2013).

In view of these advances, we first examined morphological structures of the microfibers under a Zeiss Axio Scope A1 fitted with polarizing filters and differential interference contrast (DIC) optics, at 100×, 200× and/or 400× magnifications; and images were taken using Zeiss Axiocam HRc digital cameras and Zeiss Axiovision software Version 4.9. We then tested the presence of fibrillar orientation on fibers following the method illustrated in published reference (Haugan and Holst, 2013). This part of analyses was performed with a Nikon Eclipse E200 microscope fitted with polarizers and a red tint plate (530-nm full wave compensator), and images were taken using Pixe LINK PL-D775 UC digital camera software.

Fiber morphological identifications are based on published information, as well as our modern reference collection of plant and animal fibers purchased from market (raw cotton from Organic Cotton Plus, dyed cotton yarn from BambooMN, hemp fiber from Living Dreams Vegan Yarn and Fiber, natural flax from MK Unique Designs, and wool yarn from Kondoos). It is not practical to remove specific microremains sealed on slides for ^14^C dating, and recovered microfibers occur in quantities too small to be AMS dated. Therefore, we rely on the dates obtained from the Middle Chalcolithic (all fall within the 5,200-4,700 cal BC range) materials uncovered in the same stratigraphical contexts at the site, a practice commonly employed in the studies of microparticles (such as starch, phytolith, and pollen remains) from sediments.

The laboratories at the Stanford Archaeology Center have been regularly cleaned and tested to ensure that ancient samples are not contaminated by the environment or equipment (Crowther et al., 2014). Given our strictly controlled sampling and processing procedures, in addition to the site taphonomy and very small quantities of soil samples as described above, it is highly unlikely that the abundant fibers found in the samples (see below) were significantly affected by modern contamination. It is important to note that no synthetic fibers were identified in the samples taken (sherds and soil sample alike). Since most modern clothes are made of blended or synthetic fibers, the absence of synthetic fibers is indicative of the ancient origins of fiber remains analyzed.

## 3 Results

### 3.1 Fiber remains

A total of 123 fibers were recovered from all 16 samples (**Table 1**), and identified as bast, cotton (*Gossypium* sp.), animal hair, or unidentifiable based on their morphology. Many are not identifiable to taxon (UNID; n=42; 34.1% of the total fibers retrieved) due to their lack of diagnostic features or heavy damage. Most fibers are fragmentary and/or weathered, but a few are well preserved. Such preservation is possible because fibers were trapped in small soil microaggregates, as described in Haslam (2004) study of starch.

**Figure 2:**
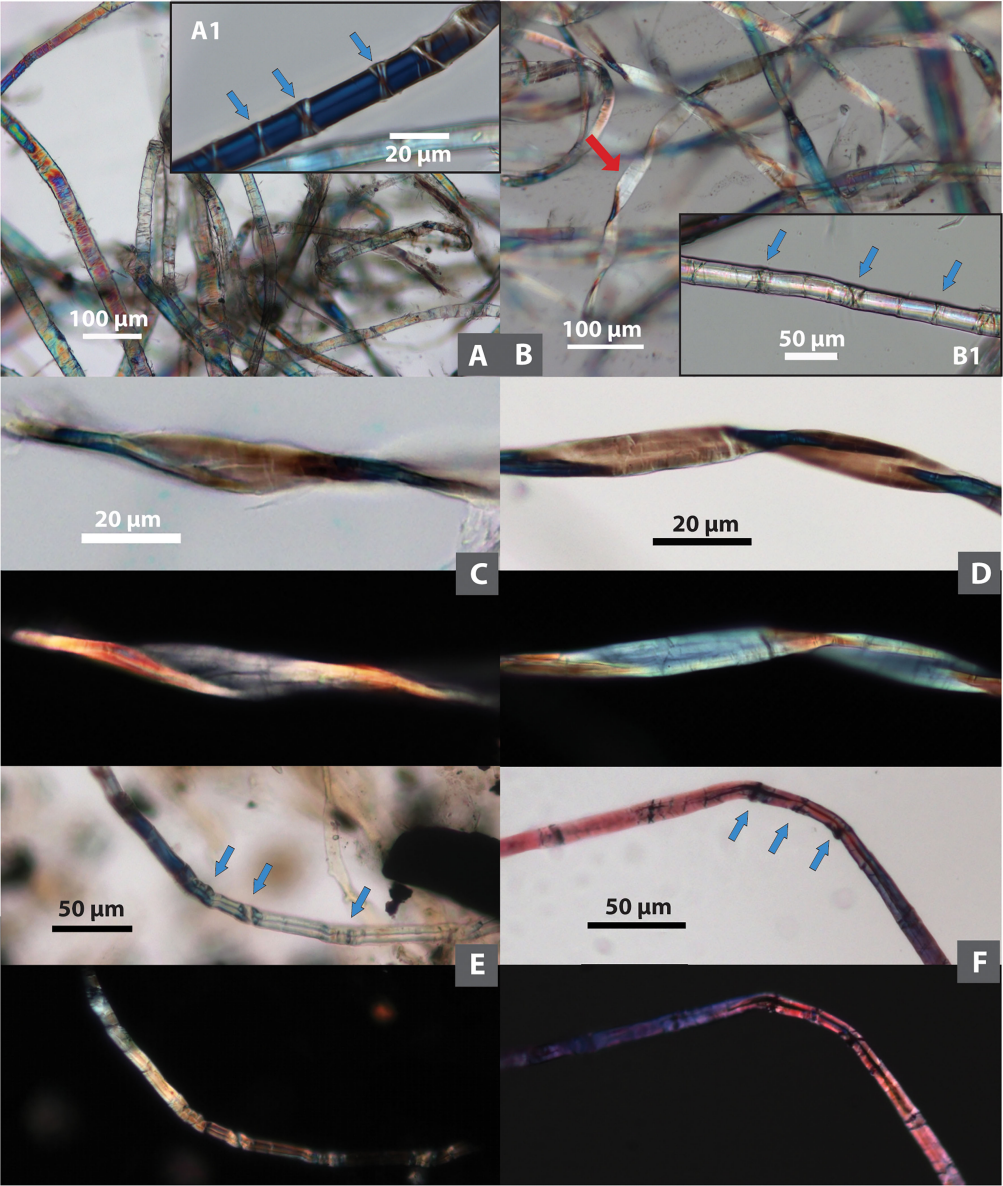
Comparison of modern and ancient bast fibers. *Modern bast fibers*: **(A)** Flax, showing segmented structure with transverse dislocations or nodes (pointed with arrows in A1); **(B)** hemp, showing segmented structure with transverse dislocations or nodes (pointed with arrows in B1); one fiber with twisted ribbon formation (pointed with arrow); **(C)** and **(D)** flax and hemp, showing a ribbon-like form with multiple transverse lines on the flattened surface. *Tel Tsaf bast fibers*: **(E)** undyed fiber; **(F)** fiber dyed in pink (**C–F** same fiber shown in brightfield and polarized views; arrows pointing to transverse dislocations).

Bast is the most frequent fiber type, found in 14 samples (n=62; 50.4% of the total; 87.5% ubiquity). The most common bast fibers in the ancient Near East include flax (*Linum*), hemp (*Cannabis*), and jute (*Corchorus*), of which only flax (the earliest one documented in the archaeological record) is considered as a native plant (Strand, 2012). Bast fiber can be identified by its segmented structure with transverse dislocations or nodes (Goodway, 1987; Bergfjord and Holst, 2010) (**Figures 2A1-B1**). In some cases, very thin bast fibers exhibit twisted ribbon forms, but they maintain visible transverse lines on the surface, as we observed on flax and hemp in our reference samples (**Figures 2B-D**), a pattern consistent with those normal fibers.

Although these morphological features can help distinguish bast from cotton and wool, it is not easy to separate different types of bast fibers, and the characteristic traits used for identification have been controversial (Bergfjord and Holst, 2010; Haugan and Holst, 2014). In this situation, the modified Herzog test is necessary.

**Figure 3:**
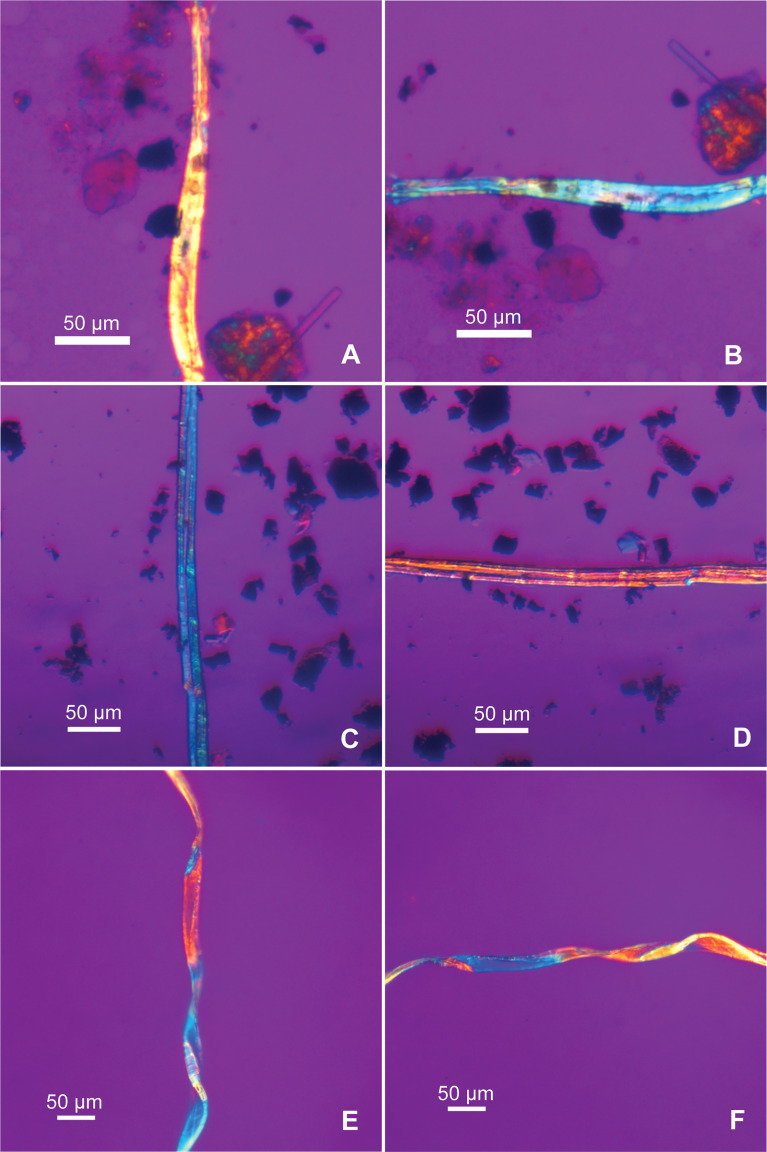
The modified Herzog test performed on Tel Tsaf fibers. To the left the sample orientation angle is 0°, to the right the sample orientation angle is 90°; **(A, B)** bast fiber showing S-twist; **(C, D)** bast fiber showing Z-twist; **(E, F)** fiber showing no well-defined fibrillar orientation, but rapid color change along the fiber, consistent with cotton.

In Tel Tsaf samples, bast fibers show small cross-section diameter (5.84-27.77 µm in thickness, with an average of 14.32 µm) and nodes forming a segmented structure (**Figures 2E, F**). When examining them with the modified Herzog test, both S-twist and Z-twist are present (**Figures 3A-D**). It is known that flax shows S-twist, while hemp and jute show Z-twist (Bergfjord and Holst, 2010: **Table 1**); thus, the bast fibers at Tel Tsaf include at least two plants. Given that flax, hemp, and jute were the common bast fibers in the ancient Near East (Strand, 2012), flax, as a local plant with S-twist fiber, would be a likely candidate, but it is not easy to pinpoint specific taxa from the Z-twist fibers, which could be hemp and/or jute. Thus, we do not identify them more specifically.

**Figure 4:**
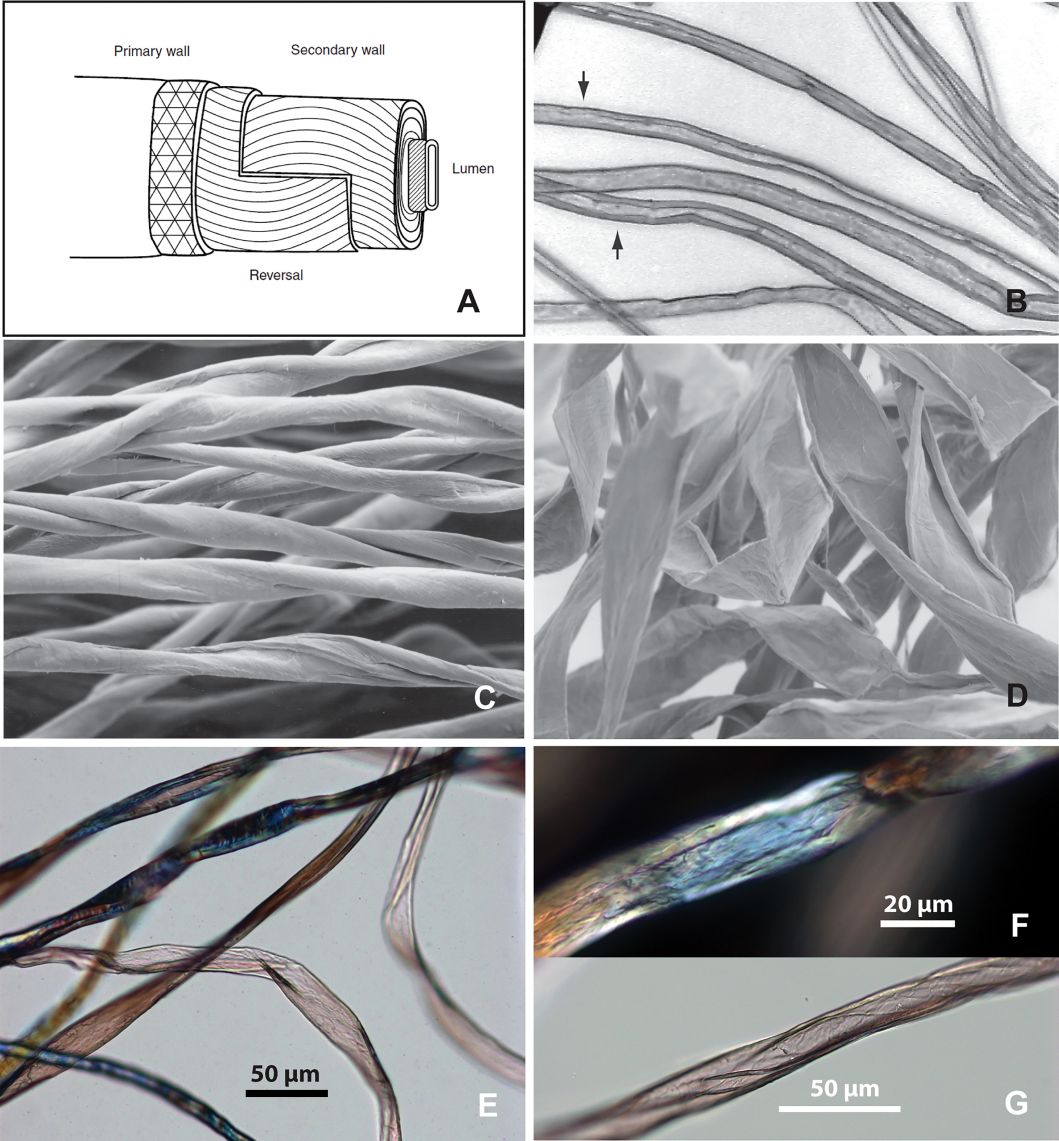
Structure and morphology of modern cotton fibers. **(A)** Multi-layered structure of cotton fiber; **(B)** mature fibers before dried, appear as long cylinders with thick cell walls (arrows); **(C)** Dried mature fibers twisted and flattened, showing the convolutions of the fibers; **(D)** Dried immature fibers form thin, flattened ribbons, and exhibit abrupt bends and flat twists (after Seagull and Alspaugh, 2001: figs. 41, 45-47); **(E)** cotton fibers showing different morphology in one sample, comparable to **B-D**; **(F)** cotton fiber showing relatively smooth surface; **(G)** cotton fiber showing oblique networking.

**Figure 5:**
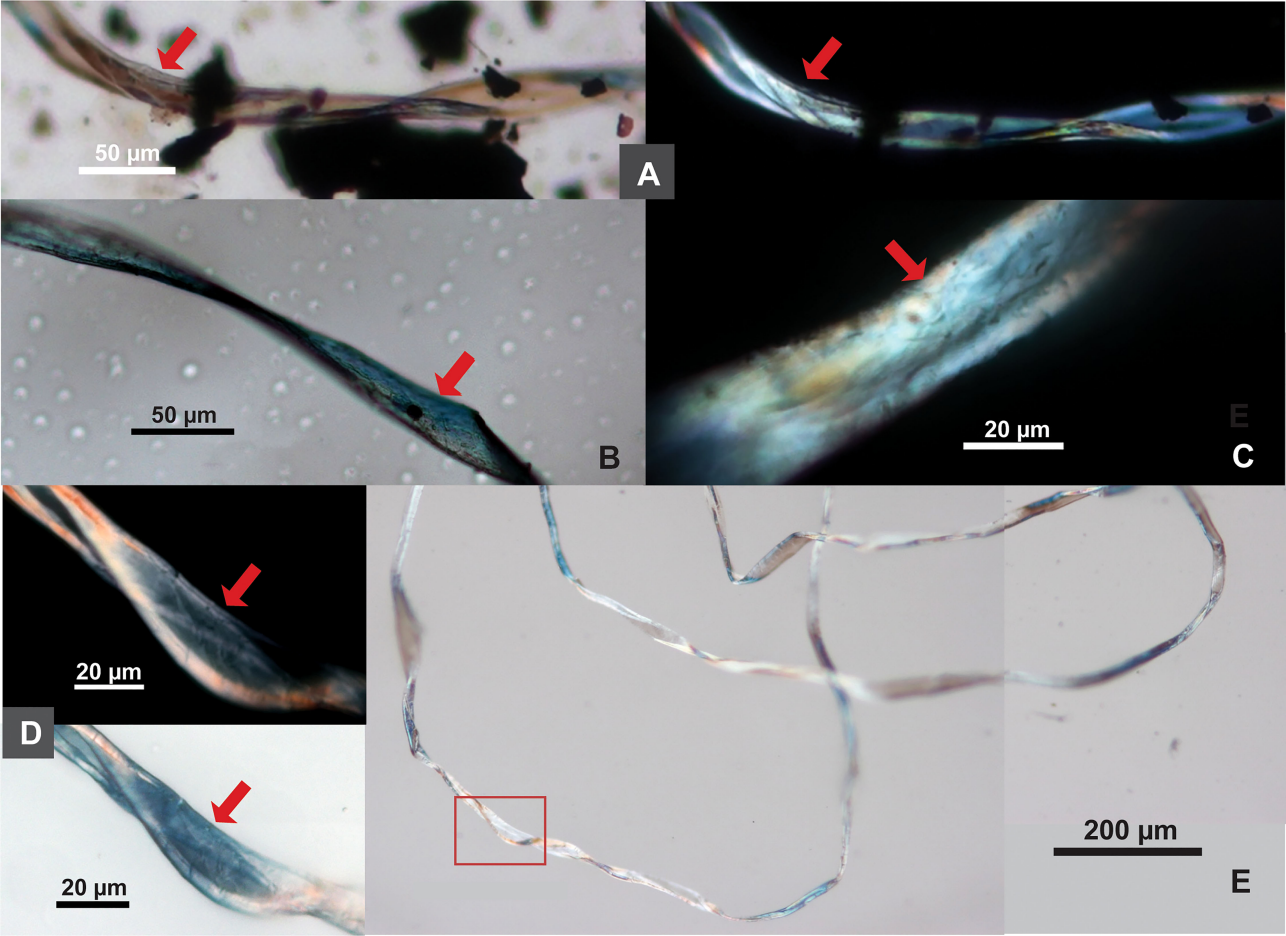
Cotton fibers from Tel Tsaf. **(A)** Fiber resembling mature cotton, twisted ribbon form with oblique networking (in brightfield and polarized views); **(B)** fiber resembling immature cotton, flattened ribbon-form, abruptly bend and twist with oblique networking; **(C)** fiber resembling mature cotton with networking patterns; **(D)** details of network pattern (in polarized and DIC views) from one location (red square) of the long fiber in E; **(E)** a long, twisted ribbon-like fiber, without transverse dislocation or nodes (red arrows pointing to network patterns).

Cotton (*Gossypium* sp.) fibers are the second most represented, identified in six samples (n=17; 13.8% of the total; 37.5% ubiquity), all found inside Room C70 (**Table 1**). A cotton fiber has a multilayered structure, which consists of a primary wall, a secondary wall and a lumen. Under the microscope, a cotton fiber looks like a twisted ribbon or a collapsed and twisted tube. Cotton fibers are normally 12-60 mm in length and 12-22 µm in diameter and exhibit some variations in morphology. The mature fiber, before it dries, appears as a long cylinder. When mature fibers dry, they twist and flatten slightly. If fibers die before sufficient secondary wall develops, they are considered to be immature; such fibers form flat ribbons, lack convolutions, and exhibit abrupt bends and flat twists (**Figures 4A-D**) (Seagull and Alspaugh, 2001:44-51). In most cases, the surface of the flattened medulla is smooth, but some show oblique networking (Kvavadze et al., 2010b: **Figure 5**). We have observed such network patterns in our reference cotton samples (**Figures 4E-G**), which clearly differ from those transverse lines present on ribbon-like bast fibers (**Figures 2C, D**). Therefore, the characteristics of the surface pattern are significant for separating cotton from bast fibers when twisted ribbon fibers are encountered in ancient samples.

In the Tel Tsaf samples, the fibers identified as cotton are characterized by a ribbon-like form and flattened medulla. Some are twisted spirally or irregularly (either clockwise or counterclockwise), the edges are thickened, resembling the mature cotton fibers. Some long ribbon-like fibers show no signs of the segmented structure that characterizes bast (**Figure 5E**). Some fibers appear as flat ribbons with abrupt bends (**Figure 5B**), similar to those immature fibers in our modern sample (**Figures 4D, E**). Some fibers show traces of networking (**Figures 5A-D**), also comparable with modern cotton samples (**Figure 4G**). The width range is 12-47 µm, which is greater than the undamaged, modern cotton sample in our database (14-27 µm; 22.42 µm on average); this size difference is probably due to damage. When the modified Herzog test was performed, these fibers exhibited rapid color change as expected for cotton described in published information (Haugan and Holst, 2013), further confirming the morphological identification (**Figures 3E, F**).

**Figure 6:**
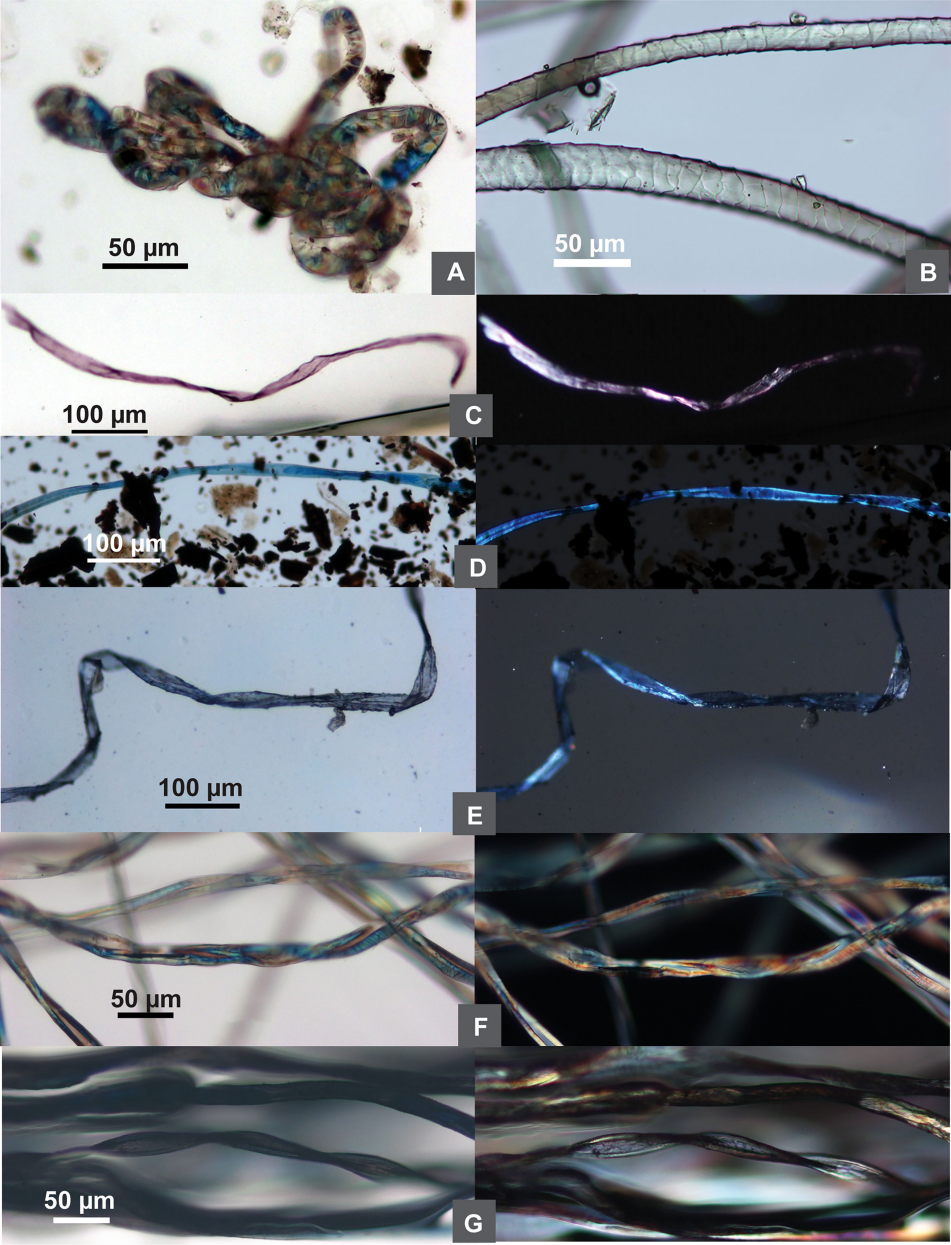
Comparison of ancient and modern wool and dyed fibers. **(A)** Wool from Tel Tsaf; **(B)** modern sheep wool; **(C–E)** dyed fibers from Tel Tsaf; **(F)** modern undyed cotton; **(G)** modern dyed black cotton; **(C–G)** each sample showing brightfield/DIC and polarized views).

Animal hairs are the least represented in the assemblage, found in two samples only (n=2; 1.6% of the total, 12.5% ubiquity). Animal hairs are characterized by a round cross-section and scaled surface patterns (Goodway, 1987). The two animal fibers in our samples are heavily worn, but their scale patterns seem to match to those of sheep’s wool when compared with published materials and our reference collection (**Figures 6A, B**). However, since only two animal fibers were present, it is uncertain if they were from woolen textiles. They are not discussed any further in this paper.

Although the UNID fibers were not assigned taxonomically, their general forms are more like plant fibers (perhaps bast) than wool. The four control samples from the surface soils yielded nine fibers in total, of which seven are identifiable as bast fibers and two are too damaged to be identified. No cotton or animal fibers were found in the control samples (**Table 1**). The number of fibers (n=1-3; 2.5 on average) in the controls are much lower than those in most of the residue samples (n=1-22; 7.7 on average). Therefore, the higher fiber densities in the residue samples suggest that these fibers were most likely *in situ*, based on comparable experimental study of microparticle movements in soil (Therin, 1998). The identifiable fiber type (bast only) in the controls also clearly differs from those in the ancient residue samples (bast, cotton, and wool). These differences between ancient and control assemblages further confirm the authenticity of fibers from residue samples. These multiple lines of evidence thus rule out the possibility of later contaminations of the ancient fiber assemblage.

### 3.2 Dyed fibers

Some fibers appear to be dyed. Natural fibers are birefringent under polarized light, and dyed and undyed fibers exhibit different colorations microscopically based on our modern reference database. Undyed natural cotton and bast fibers, if not damaged, normally appear polychromatic in both brightfield and polarized views, the colors of the same fibers in the two microscopic views do not necessarily match one another (**Figures 2A-D**, **6F**). These phenomena have been found in some cotton and bast fibers from Tel Tsaf, indicating their undyed conditions (**Figures 2E**, **5A, D, E**). When fibers are dyed, the same color is almost uniformly exhibited in both brightfield and polarized views, also closely resembling the color on actual dyed fiber (**Figure 6G**). Thus, these characteristics were used here to distinguish dyed and undyed fibers and infer their original colors. As a result, 24 dyed fibers were found in the assemblage (from bast, cotton and UNID), and colors include blue (n=16), pink (n=3), purple (n=1), green (n=1), and brown/black (n=3) (**Figures 2F**, **5B**, **6C-E**).

## 4 Discussion

### 4.1 Authenticity of microbotanical remains

Microbotanical remains, such as plant fibers, starch, and pollen, have often been recovered from deep archaeological sediments, and their age, mechanisms of preservation, and authentic connections with anthropogenic materials have been the subject of research (Haslam, 2004). There is a lack of quantitative study of the diachronic degradation of archaeological fiber microremains. Plant fibers are generally composed of cellulose, which is made of glucose molecules, like starch, and can be degraded by enzymatic activities from fungi and bacteria in the environment (Kuhad et al., 1997; Haslam, 2004). The conditions resulting in the longevity of starch remains in sediments have been investigated and can be used here as references. Starch granules associated with small soil microaggregates (<53-250 µm) tend to survive much longer than those associated with macroaggregates (>250 µm) in sediments; this is because the organic materials in the former condition are less subjected to fungi and bacteria-induced enzymatic activities (Guggenberger et al., 1999; Haslam, 2004). Studies of downward movements of starch in sediments similar to wet tropical environments with four meters of rain per year indicate that starch grains move under the influence of groundwater. However, only smaller grains (0-5 µm in size) travel more freely, while large grains generally do not move notable distances. These studies thus concluded that the high frequency of starch grains found in archaeological sediments and on artifacts is not the result of large scale movement (Therin, 1998; Haslam, 2004). In addition, the survival of microfibers in sediments is also related to soil type. It is known that the pH value of soil affects preservation of animal and vegetal fibers differently: vegetal fibers survive better in alkaline conditions (above 7.0), whereas protein-based animal fibers, such as wool, preserve better in slightly acidic environments (below 7.0) (Cybulska and Maik, 2007).

These studies indicate that microfossils, including fibers, found in archaeological deposits are not necessarily the results of post-depositional contamination due to downward movements of microremains in the sediments, but careful analysis of taphonomic processes is needed to exclude possible later intrusions.

In the current study, abundant vegetal fibers found in anthropogenic sediments at Tel Tsaf may be attributable to two main factors. First, the site was a locality of intensive human activities with rich macro and micro material remains. Second, the soil at the site is alkaline, with a pH value range of 7.71–8.06 (Hubbard, 2015: table 3.29), and the alkaline soils are likely to help the preservation of vegetal fibers as discussed earlier (Cybulska and Maik, 2007). From here, we focus exclusively on the vegetal fibers recovered.

### 4.2 Multiple bast fiber textiles in the southern Levant

The history of textile production in this region goes hand in hand with advances in the domestication of plants and animals and the development of specific tools for cutting and processing fibers. The earliest concrete evidence for textiles is dated to the Pre-Pottery Neolithic B at Nahal Hemar Cave in Israel (ca. 7,700–7,100 cal BC), where fragments of flax textiles were discovered (Schick, 1988). Stone items reflecting textile production (such as spindle whorls) appear in the record during the advent of farming communities in the region, as early as the Pre-Pottery Neolithic period C (ca. 7,000–6,400 cal BC) or slightly earlier and are made of mainly limestone (Rosenberg and Garfinkel, 2014). Later, with the first appearance of pottery in the southern Levant, in the context of the Yarmukian culture of the Pottery Neolithic period, these whorls are also made of clay, either reworked sherds or specifically made clay whorls (Rosenberg and Garfinkel, 2014). Indeed, most Neolithic and Chalcolithic fabrics and textiles from the southern Levant have been reported as flax-made (Bar-Adon, 1980; Schick, 1998; Shamir, 2014; Langgut et al., 2016). Thus, flax has been regarded as the dominant material of textiles until the emergence of wool textiles, probably around the Middle Bronze Age, ca. 1,950–1,500 cal BC (Shamir, 2014; Shamir, 2015; Shamir and Schick, 2019). This view, however, is now questioned by the bast fibers showing both S-twist and Z-twist fibrillar orientations in the Tel Tsaf assemblage. The current finding suggests that multiple bast fiber plants, including not only flax, but also hemp and/or jute, may have already present by the Middle Chalcolithic in the region.

### 4.3 The earliest cotton

The cotton fibers found at Tel Tsaf are the earliest known evidence of this non-indigenous plant in the Near East (**Figure 1A**). Today there are four cultivated cotton species in the world. Two of these are of South and Central American origins, whereas the other two are of the Old World. The two Old World cotton species, both closely related diploids, are *Gossypium arboretum* from the Indian subcontinent and *Gossypium herbaceum* from Africa, which were domesticated independently as demonstrated by DNA analysis (Fuller, 2008; Renny-Byfield et al., 2016; Viot, 2019).

The Indus Valley has been suggested as a possible center of domestication and diffusion of *G. arboreum* (Fuller, 2008). The earliest known evidence for actual cotton fibers comes from the Aceramic Neolithic period I at the Mehrgarh burial site in central Balochistan, Pakistan. Here, cotton fiber threads used to string copper beads were recovered, dating to the first half of the 6^th^ millennium BC (Moulherat et al., 2002), roughly within the range of 6,000-5,500 BC. The earliest known cotton fabric is a small fragment of cloth adhering to the lid of a small silver vase found at Mohenjo-daro, dating to 3,000-2,750 BC (Gulati and Turner, 1929). Based primarily on evidence from seeds (Fuller, 2008), cotton domestication is hypothesized to have occurred during the time of the Harappan civilization (2,600-1,900 BC).

Archaeological cotton records in Africa are much later than those in South Asia. At least 15 sites in northeastern Africa and western Arabia have revealed cotton remains, dating from the 1^st^ century BC to the 7^th^ century AD (Bouchaud et al., 2018). Ancient DNA analyses conducted on cotton seeds from Qasr Ibrim, Egyptian Nubia (4^th^ century AD; **Figure 1A**) have determined the species to be *G. herbaceum*, which helps to clarify the African origin of ancient Egyptian cotton (Palmer et al., 2012).

In the Near East, the earliest cotton remains were previously reported from Dhuweila, in the Badia of eastern Jordan, where fibers and impressions of a woven cotton fabric were found on the surface of lime plaster, dating to a context of the Late Chalcolithic or Early Bronze Age (ca. 4,450-3,000 BC). Since the site does not lie on any important transportation routes, it was suggested that these fabric fragments found their way to the site through casual visits by nomadic hunters or shepherds (Betts et al., 1994). Microscopic cotton fibers have also been recovered from the burial contexts at Saphar-Kharaba, southern Georgia, dating to the Late Bronze Age (15^th^-14^th^ centuries BC), where people used mainly flax and cotton textiles for funerary clothing and other burial materials. The presence of cotton in this region was attributed to trade relations between the southern Caucasus and the Indus valley (Kvavadze et al., 2010b). Later, the evidence for cotton as a cultivated fiber and woven textile was found at a royal Assyrian burial at Nimrud in modern day northern Iraq, dating to the 8^th^ century BC (Toray Industries, Inc, 1996; Álvarez-Mon, 2015) (**Figure 1A**).

These findings show that the actual cotton textiles appeared in the archaeological record in Iraq about 500 years later than cotton microfibers as remnants of textiles in Georgia, which is further away from the source of cotton domestication in the Indus valley. Thus, cotton products may have been moved around through trade, exchange, or casual contacts by people long before evidence for the presence of actual textiles in the archaeological record. The cotton remains found at Dhuweila exemplify such an early movement. Notably, Tel Tsaf, ca. 200 km west of Dhuweila (**Figure 1A**), was located within a trade network and more likely to have engaged to long-distance communications. Also, in addition to cotton fibers, many artifacts with foreign origins (e.g., an Ubaid sherd, obsidian and olivine beads, tokens, figurines, and a copper awl) were found inside or near Room C70 at Tel Tsaf (**Figures 1G-I**) (Garfinkel et al., 2014; Rosenberg and Klimscha, 2021; Rosenberg et al., 2022a; Rosenberg et al., 2022b). This room appears to have been a locality of intensive activities with people associated with long-distance exchange.

The well dated cotton fibers found at Tel Tsaf were several hundred years later than the cotton strings from the Mehrgarh copper beads, but at least 300 years earlier than the cotton fabric from Dhuweila in Jordan. These cotton remains are likely from a wild species, as they predate the presumed cotton domestication date, as determined by seeds from the Harappa civilization in the Indus valley for some two millennia (Fuller, 2008). As so far there is no evidence of cotton cultivation in the Levant during the Chalcolithic period, the cotton fibers found at Tel Tsaf must have come from one of the possible Old World source locations (Indus valley or Africa). The cotton finds from Africa are much later than those of Tel Tsaf, whereas the cotton remains from Tel Tsaf and Dhuweila are in spatial proximity. The current evidence thus supports an Indian subcontinent source for the Tsaf cotton, similar to what has been suggested for Dhuweila (Betts et al., 1994).

### 4.4 Dyed fibers

Natural dyes used to color fibers are generally soluble organic compounds that can be extracted from plants, parasitic insects, and secretions of a sea snail. Many species of plants were widely used for dyes around world. For example, indigo blue, one of the earliest blue dyes, was made from plant indigo (*Indigofera tinctoria*), possibly originated in ancient South Asia; and the most common yellow dye was obtained from safflower (*Carthamus tinctorius*), which was grown in Egypt and Iran (Ahmed, 2009; Melo, 2009). The archaeological and textural records have revealed that dyeing textiles and threads began at a very early date and developed with an increasing range of colors, techniques and color sources (Barber, 1991; Abel, 2012:223-243; Melo, 2009). The earliest known examples have been traced to the Upper Paleolithic times. For example, the remains of black, gray, turquoise, and pink colored bast fibers were found in the 30,000-year-old deposits at Dzudzuana in Georgia (Kvavadze et al., 2009). Likewise, at the Upper Paleolithic site of Shizitan Locality 29 in north China, significant numbers of dyed bast and wool fibers have been identified from depositional layers 1-7, measuring more than 11 meters in depth (ca. 26,000-13,500 cal. BP); and the colors include blue, gray, pink, brown, and black (Song et al., 2017). In the Near East, dyed threads were reported from the early Neolithic site of Çatalhöyük in Turkey (ca. 9,400-8,200 cal. BP), where Mellaart found a number of broken beads with traces of red inside the string holes (Mellaart, 1967:219). In the Levant, a scrap of red and perhaps also green woolen cloth was found in the Late Chalcolithic Cave of the Treasure at Nahal Mishmar, Israel (Bar-Adon, 1980). These examples indicate that dyeing technology was developed long before the Chalcolithic period, and this decorative method may have commonly applied to strings and textiles in many parts of the world. The fibers dyed in multiple colors at Tel Tsaf may have come from people’s colorful clothing or threads for stringing beads, among other objects.

## 5 Conclusions

Microparticle analyses have long been employed in the study of pollen, phytolith, starch granules, and other materials, but few attempts have been made to investigate microfibers in archaeological contexts. The rarity of actual textile remains in the archaeological record has affected our understanding of an important aspect of human history related to ancient technologies and social interactions. The current research demonstrates the effectiveness of microfiber analysis, which has only been sporadically employed previous for overcoming the preservation problems with fibers and textiles. The presence of a substantial number of prehistoric microfibers from anthropogenic sediments at Tel Tsaf, together with similar remains previously reported from a few sites in Israel and Georgia, indicates the great potential for identifying archaeological fibers, which has been largely overlooked in most excavation and sampling strategies.

Tel Tsaf was an important settlement, which played a pivotal role in trans-regional interaction networks during the Middle Chalcolithic period. A variety of non-local material items found at the site point to its connections with remote social groups in all directions. The new evidence of multi-color cotton and bast fibers in living areas add another dimension to the complex social activities at the settlement. If Tel Tsaf was a part of larger trade networks, the presence of some non-local goods may have been associated with movements of people from far away. The uses of non-local fibers at the site, for example from travelers’ clothing or belongings, would be an interesting research topic for future studies.

The new finds from Tel Tsaf place the appearance of cotton in the Near East several hundred years earlier than previously thought, thus shedding new light on trans-regional interactions, bridging the Levant and South Asia during the early phases of increasing social and economic complexity in this part of the world. It is not possible to determine if the cotton from Tel Tsaf was wild or domesticated based on microremain analysis. Nevertheless, if some of these cotton fibers were of textiles or strings originated in Indus Valley during the early fifth millennium BC, one would have to ask if the timing of cotton domestication in its source may have been earlier than the third millennium BC as previously suggested, or if wild cotton products were more prevalent than previously thought. The finding of bast fibers likely including flax and other plants also sheds new light on the diverse fiber technologies in the Chalcolithic Near East.

## Data availability statement

The original contributions presented in the study are included in the article/supplementary material. Further inquiries can be directed to the corresponding author.

## Author contributions

LL and DR contributed to conception and design of the study. DR and FK excavated the site and collected samples. LL and ML analyzed the data. LL, DR, and ML wrote the manuscript. All authors contributed to the article and approved the submitted version.

## References

[B1] Abel A. (2012). “The history of dyes and pigments,” in Colour design: Theories and applications. Ed. Best J. (Cambridge: Woodhead Publishing), 557–587.

[B2] Ahmed H. E. (2009). “History of natural dyes in north Africa ‘Egypt’,” in Handbook of natural colorants. Eds. Bechtold T. Mussak R. (West Sussex: Wiley), 27–36.

[B3] Álvarez-Mon J. (2015). The introduction of cotton into the near East: A view from elam. Int. J. Soc. Iranian Archaeol. 1 (2), ­41–52.

[B4] Bar-Adon P. (1980). The cave of the treasure: the finds from the caves in nahal mishmar (Jerusalem: Israel Exploration Society).

[B5] Barber E. J. W. (1991). Prehistoric textiles (Cambridge: Cambridge University Press).

[B6] Bar-Yosef O. (2020). “The neolithic revolution in the fertile crescent and the origins of fibre technology,” in The competition of fibres: Early textile production in Western Asia, south-east and central Europe (10,000-500 BC). Eds. Schier W. Pollock S. (Oxford and Philadelphia: Oxbow).

[B7] Becker C. Benecke N. Grabundzija A. Küchelmann H.-C. Pollock S. Schier W.. (2016). The textile revolution. research into the origin and spread of wool production between the near East and central Europe. J. Ancient Stud. 6, 102–151. doi: 10.17169/FUDOCS_document_000000025988

[B8] Bergfjord C. Holst B. (2010). A procedure for identifying textile bast fibres using microscopy: Flax, nettle/ramie, hemp and jute. Ultramicroscopy 110, 1192–1197. doi: 10.1016/j.ultramic.2010.04.014 20462699

[B9] Betts A. Borg K. d. A. McClintock C. Strydonck M. V. (1994). Early cotton in north Arabia. J. Archaeol. Sci. 21, 489–499. doi: 10.1006/jasc.1994.1049

[B10] Bouchaud C. Clapham A. Newton C. Tallet G. Thanheiser U. (2018). “Cottoning on to cotton (Gossypium spp.) in Arabia and Africa during antiquity,” in Plants and people in the African past: Progress in African archaeobotany. Eds. Mercuri A. M. D’Andrea A. C. Fornaciari R. Höhn A. (Springer: Springer International Publishing).

[B11] Breniquet C. (2020). “Early wool of Mesopotamia, c. 7000–3000 BC. between prestige and economy,” in The competition of fibres: Early textile production in Western Asia, south-east and central Europe (10,000-500 BC). Eds. Schier W. Pollock S. (Oxford and Philadelphia: Oxbow), 17–26.

[B12] Crowther A. Haslam M. Oakden N. Walde D. Mercader,. J. (2014). Documenting contamination in ancient starch laboratories. J. Archaeol. Sci. 49, 90–104. doi: 10.1016/j.jas.2014.04.023

[B13] Cybulska M. Maik J. (2007). Archaeological textiles–a need for new methods of analysis and reconstruction. Fibers Textiles Eastern Europe 15 (5-6), 185–189.

[B14] Freikman M. Ben-Shlomo D. Garfinkel Y. (2021). A stamped sealing from middle chalcolithic tel tsaf: implications for the rise of administrative practices in the Levant. Levant 53 (1), 1–12. doi: 10.1080/00758914.2021.1923906

[B15] Fuller D. Q. (2008). “The spread of textile production and textile crops in India beyond the harappan zone: an aspect of the emergence of craft specialization and systematic trade,” in Linguistics, archaeology and the human past. Eds. Osada T. Uesugi A. (Kyoto: Research Institute for Humanity and Nature), 1–26.

[B16] Garfinkel Y. Ben-Shlomo D. Freikman M. (2020). Excavations at tel tsaf 2004–2007: Final report Vol. 1 (Ariel: Ariel University Press).

[B17] Garfinkel Y. Freikman M. Ben-Shlomo D. Vered A. (2007). Tel tsaf: The 2004-2006 excavation seasons. Israel Explor. J. 57 (1), 1–33.

[B18] Garfinkel Y. Klimscha F. Shalev S. Rosenberg D. (2014). The beginning of metallurgy in the southern Levant: a late 6th millennium CalBC copper awl from tel tsaf, Israel. PloS One 9 (3), e92591. doi: 10.1371/journal.pone.0092591 24671185 PMC3966803

[B19] Garfınkel Y. Ben-Shlomo D. Kuperman T. (2009). Large-Scale storage of grain surplus in the sixth millennium BC: the silos of tel tsaf. Antiquity 83, 309–325. doi: 10.1017/S0003598X00098458

[B20] Gilligan I. (2010). The prehistoric development of clothing: Archaeological implications of a thermal model. J. Archaeol. Method Theory 17 (1), 15–80. doi: 10.1007/s10816-009-9076-x

[B21] Goodway M. (1987). Fiber identification in practice. J. Am. Institute Conserv. 26 (1), 27–44. doi: 10.1179/019713687806027933

[B22] Guggenberger G. Elliott E. T. Frey S. D. Six J. Paustian K. (1999). Microbial contributions to the aggregation of a cultivated grassland soil amended with starch. Soil Biol. Biochem. 31, 407–419. doi: 10.1016/S0038-0717(98)00143-6

[B23] Gulati A. N. Turner A. J. (1929). 1–a note on the early history of cotton. J. Textile Institute Trans. 20 (1), T1–T9. doi: 10.1080/19447022908661470

[B24] Hardy B. L. Moncel M. H. Kerfant C. Lebon M. Bellot-Gurlet L. Melard N. (2020). Direct evidence of Neanderthal fibre technology and its cognitive and behavioral implications. Sci. Rep. 10 (1), 4889. doi: 10.1038/s41598-020-61839-w 32273518 PMC7145842

[B25] Haslam M. (2004). The decomposition of starch grains in soils: implications for archaeological residue analyses. J. Archaeol. Sci. 31 (12), 1715–1734. doi: 10.1016/j.jas.2004.05.006

[B26] Haugan E. Holst B. (2013). Determining the fibrillar orientation of bast fibres with polarized light microscopy: the modified herzog test (red plate test) explained. J. Microsc. 252 (2), 159–168. doi: 10.1111/jmi.12079 24020614 PMC4263192

[B27] Haugan E. Holst B. (2014). Flax looking-alines: Pitfalls of ancient plant fibre identification. Archaeometry 56 (6), 951–960. doi: 10.1111/arcm.12054

[B28] Henry A. G. (2020). Handbook for the analysis of micro-particles in archaeological samples (New York City: Springer).

[B29] Hubbard E. M. (2015) A geoarchaeological investigation of storage and surplus at tel tsaf, Israel (Toronto: University of Toronto).

[B30] Hurcombe L. (2014). Perishable material culture in preshistory: Investigating the missing majority (London and New York: Routledge).

[B31] Juhola T. Henry A. G. Kirkinen T. Laakkonen J. Väliranta M. (2019). Phytoliths, parasites, fibers, and feathers from dental calculus and sediment from iron age luistari cemetery, Finland. Quaternary Sci. Rev. 222, 105888. doi: 10.1016/j.quascirev.2019.105888

[B32] Kuhad R. C. Singh A. Eriksson K.-E. (1997). Microorganisms and enzymes involved in the degradation of plant fiber cell walls. Adv. Biochem. Engineering/Biotechnol. 57, 45–125. doi: 10.1007/BFb0102072 9204751

[B33] Kvavadze E. Bar-Yosef O. Belfer-Cohen A. Boaretto E. Jakeli N. Matskevich Z.. (2009). 30,000-Year-Old wild flax fibers. Science 325, 1359. doi: 10.1126/science.1175404 19745144

[B34] Kvavadze E. Bar-Yosef O. Belfer-Cohen A. Boaretto E. Jakeli N. Matskevich Z.. (2010a). Response to comment on “30,000-Year-Old wild flax fibers”. Science 328, 1634. doi: 10.1126/science.1187161 19745144

[B35] Kvavadze E. Narimanishvili G. Bitadze L. (2010b). Fibres of *Linum* (flax), *Gossypium* (cotton) and animal wool as non-pollen palynomorphs in the late bronze age burials of saphar-kharaba, southern Georgia. Vegetation History Archaeobot. 19 (5-6), 479–494. doi: 10.1007/s00334-010-0270-2

[B36] Langgut D. Yahalom-Mack N. Lev-Yadun S. Kremer E. Ullman M. Davidovich U. (2016). The earliest near Eastern wooden spinning implements. Antiquity 90 (352), 973–990. doi: 10.15184/aqy.2016.99

[B37] Liu L. Wang J. Rosenberg D. Zhao H. Lengyel G. Nadel D. (2018). Fermented beverage and food storage in 13,000 y-old stone mortars at raqefet cave, Israel: Investigating natufian ritual feasting. J. Archaeol. sci.: Rep. 21, 783–793. doi: 10.1016/j.jasrep.2018.08.008

[B38] Lukesova H. Holst B. (2020). Is cross-section shape a distinct feature in plant fibre identification? Archaeometry 63 (1), 216–226. doi: 10.1111/arcm.12604

[B39] McCorriston J. (1997). Textile extensification, alienation, and social stratification in ancient Mesopotamia. Curr. Anthropol. 38 (4), 517–535. doi: 10.1086/204643

[B40] Mellaart J. (1967). Çatal hüyük: A neolithic town in Anatolia (New York: McGraw-Hill Book Company).

[B41] Melo M. J. (2009). “History of natural dyes in the ancient Mediterranean world,” in Handbook of natural colorants. Eds. Bechtold T. Mussak R. (West Sussex: Wiley), 3–20.

[B42] Moulherat C. Tengberg M. Haquet J.-F. Mille B. (2002). First evidence of cotton at neolithic mehrgarh, Pakistan: Analysis of mineralized fibres from a copper bead. J. Archaeol. Sci. 29, 1393–1401. doi: 10.1006/jasc.2001.0779

[B43] Nadel D. Danin A. Werker E. Schick T. Kislev M. E. Stewart K. (1994). 19,000-Year-Old twisted fibers from ohalo II. Curr. Anthropol. 35 (4), 451–458. doi: 10.1086/204303

[B44] Nosch M.-L. Koefoed H. Strand E. A. (2013). Textile production and consumption in the ancient near East: archaeology, epigraphy, iconography (Oxford: Oxbow Books).

[B45] O’Keefe J. M. K. Marret F. Osterloff P. Pound M. J. Shumilovskikh L. (2021). Why a new volume on non-pollen palynomorphs? Vol. 511 (Geological Society, London: Special Publications), 1–11. doi: 10.1144/sp511-2021-83

[B46] Palmer S. A. Clapham A. J. Rose P. Freitas F. O. Owen B. D. Beresford-Jones D.. (2012). Archaeogenomic evidence of punctuated genome evolution in gossypium. Mol. Biol. Evol. 29 (8), 2031–2038. doi: 10.1093/molbev/mss070 22334578

[B47] Paterson R. A. Lowe B. J. Smith C. A. Lord J. M. Ngarimu-Cameron R. (2017). Polarized light microscopy: An old technique casts new light on māori textile plants. Archaeometry 59 (5), 965–979. doi: 10.1111/arcm.12281

[B48] Rast-Eicher A. Karg S. Jørgensen L. B. (2021). The use of local fibres for textiles at neolithic Çatalhöyük. Antiquity 95 (383), 1129–1144. doi: 10.15184/aqy.2021.89

[B49] Renny-Byfield S. Page J. T. Udall J. A. Sanders W. S. Peterson D. G. Arick M. A.. (2016). Independent domestication of two old world cotton species. Genome Biol. Evol. 8 (6), 1940–1947. doi: 10.1093/gbe/evw129 27289095 PMC4943200

[B50] Rosenberg D. Elkayam Y. Garfinkel Y. Klimscha F. Vuckovic V. Weiss Y. (2022a). Long-distance trade in the middle chalcolithic of the southern Levant: The case of the olivine beads from tel tsaf, Jordan valley, Israel. PloS One 17 (8), e0271547. doi: 10.1371/journal.pone.0271547 35947578 PMC9365180

[B51] Rosenberg D. Garfinkel Y. (2014). The groundstone industry of sha’ar hagolan – stone working at the dawn of pottery production in the southern levant. Jerusalem (Hebrew University: The Institute of Archaeology, the Hebrew University of Jerusalem and the Israel Exploration Fund).

[B52] Rosenberg D. Klimscha F. (2021). Social complexity and ancient diet – the renewed research project at tel tsaf, Jordan valley. Qadmoniyut 161, 12–17.

[B53] Rosenberg D. Rizzuto B. C. Klimscha F. Carter T. (2022b). The obsidian beads from middle chalcolithic tel tsaf (ca. 5,200–4,700 cal. BC), Jordan valley, Israel: technology, provenance, and socio-economic significance. Archaeol. Anthropol. Sci. 14 (6), 113. doi: 10.1007/s12520-022-01570-x

[B54] Rots V. Hardy B. L. Serangeli J. Conard N. J. (2015). Residue and microwear analyses of the stone artifacts from schöningen. J. Hum. Evol. 89, 298–308. doi: 10.1016/j.jhevol.2015.07.005 26387038

[B55] Sabatini S. Bergerbrant S. (Eds.) (2020). The textile revolution in bronze age europe; production, specialisation, consumption (Cambridge: Cambridge University Press).

[B56] Schick T. (1988). Nahal hemar cave: cordage, basketry and fabrics. Atiqot 38, 31–43.

[B57] Schick T. (1998). The cave of the warrior: a fourth millennium burial in the judean desert Vol. 5 (Jerusalem: Israel Antiquities Authority).

[B58] Schier W. Pollock S. (Eds.) (2020). The competition of fibres: Early textile production in Western Asia, south-east and central Europe (10,000-500 BC) (Oxford and Philadelphia: Oxbow).

[B59] Seagull R. Alspaugh P. (Eds.) (2001). Cotton fiber development and processing: An ilustrated overview (Texas Tech University: International Textile Center).

[B60] Shamir O. (2014). “Textiles, basketry and other organic artifacts of the chalcolithic period in the southern Levant,” in Masters of fire: Copper age art from Israel. Eds. Brandel O. Saban M. Master D. (New York: New York University), 139–152.

[B61] Shamir O. (2015). Textiles from the chalcolithic period, early and middle bronze age in the southern Levant. Archaeol. Textile Rev. 57, 12–25.

[B62] Shamir O. Schick T. (2019). “Chalcolithic to medieval textiles from the judean desert caves survey,” in Sea And desert: On kings, nomads, cities and monks essays in honor of Joseph patrich. Eds. Peleg-Barkat O. Ashkenazi J. Leibner U. Aviam M. Between R. T. (Ostracon:Land of Galilee 5), 195–224.

[B63] Sherratt A. (1983). The secondary exploitation of animals in the old world. World Archaeol. 15 (1), 90–104. doi: 10.1080/00438243.1983.9979887

[B64] Shumilovskikh L. S. Geel B. V. (2020). “Non-pollen palynomorphs,” in Handbook for the analysis of micro-particles in archaeological samples. Ed. Henry A. G. (New York City: Springer), 65–96.

[B65] Song Y. Cohen D. J. Shi J. Wu X. Kvavadze E. Goldberg P.. (2017). Environmental reconstruction and dating of shizitan 29, shanxi province: An early microblade site in north China. J. Archaeol. Sci. 79, 19–35. doi: 10.1016/j.jas.2017.01.007

[B66] Strand E. A. (2012). The textile chaîne opératoire: using a multidisciplinary approach to textile archaeology with a focus on the ancient near East. Paléorient 38 (1-2), 21–40.

[B67] Suomela J. A. Vajanto K. Räisänen R. (2017). Seeking nettle textiles – utilizing a combination of microscopic methods for fibre identification. Stud. Conserv. 63 (7), 412–422. doi: 10.1080/00393630.2017.1410956

[B68] Therin M. (1998). “The movement of starch grains in sediments,” in A close look: Recent Australian studies of stone tools. Ed. Fullagar R. (Sydney: Sydney University Archaeological Methods Series 6), 61–72.

[B69] Toray Industries, Inc (1996). Report on the analyses of textiles uncovered at the nimrud tomb-chamber. Al-Rāfidān 17, 199–206.

[B70] Viot C. (2019). Domestication and varietal diversification of old world cultivated cottons (Gossypium sp.) in the antiquity. Rev. d’ethnoécologie (Cotton Old World: Domestication cultivation uses exchange) 15, 34–61. doi: 10.4000/ethnoecologie.4404

[B71] Zohary D. Hopf M. Weiss E. (2012). Domestication of plants in the old world. 4th ed (Oxford: Oxford University Press).

